# TFFBN-HDLF: a hybrid deep learning framework based on time-frequency functional brain networks for epileptic seizure detection

**DOI:** 10.3389/fmed.2026.1788516

**Published:** 2026-03-17

**Authors:** Peipei Gu, Ruibo Wang, Yisheng Lin, Ming Zhang, Fangqin Liu, Jiayang Guo, Bin Jiang

**Affiliations:** 1 School of Software Engineering, Zhengzhou University of Light Industry, Zhengzhou, China; 2 Department of Physics, College of Physical Science and Technology, Xiamen University, Xiamen, China; 3 Department of Pediatrics, Zhongshan Hospital Xiamen University, Xiamen, China; 4 Xiamen Shuimu Ruizhi Intelligent Technology Co., Ltd., Xiamen, China; 5 Department of Neurology and Department of Neuroscience, The First Affiliated Hospital, School of Medicine, Xiamen University, Xiamen, China; 6 Xiamen Medical Quality Control Center for Neurology, Xiamen, China

**Keywords:** attention mechanism, deep learning, EEG-based seizure detection, geriatric epilepsy, time–frequency functional brain network, transformer

## Abstract

**Introduction:**

The detection of epilepsy seizures in the elderly based on electroencephalogram (EEG) is the foundation of an intelligent clinical decision support system. However, due to the often slow background activity and complex non-stationary dynamic characteristics of the brain signals in elderly patients, existing methods often struggle to extract robust discriminative features across different individuals. To address this deficiency, this study proposes a hybrid deep learning framework named TFFBN-HDLF, aiming to enhance the reliability and diagnostic accuracy of artificial intelligence-assisted monitoring of epilepsy seizures in the elderly.

**Methods:**

Firstly, this paper presents a time-frequency functional brain network construction method (TFFBNC). By combining the Pearson correlation coefficient (PCC) and phase lag index (PLV), we construct a two-dimensional time-frequency fused functional brain network (TFPPNet). This method can comprehensively simulate the synchronous neural interactions in the time and frequency domains of the elderly brain, converting the complex raw EEG data into high-quality neurophysiological evidence, thereby providing a basis for clinical decision-making. Additionally, we have developed a hybrid deep learning architecture-SeizureTransNet, which combines convolutional neural networks (CNNs) with enhanced Transformer modules. This architecture can dynamically select and integrate multi-scale spatiotemporal features, ensuring accurate inference of the seizure state in the elderly while maintaining high adaptability to the different EEG pattern differences caused by aging.

**Results:**

Extensive evaluations on publicly available CHB-MIT and Siena datasets have confirmed the effectiveness of this framework. The accuracy of TFFBN-HDLF on the CHB-MIT dataset reached 98.09% (AUC of 99.45%), and on the Siena dataset, it was 92.49% (AUC of 95.64%).

**Discussion:**

These results indicate that the collaborative integration of attention-based time-frequency network fusion and feature learning significantly improves diagnostic performance, demonstrating its potential application in clinical care for epilepsy in the elderly.

## 1 Introduction

Epilepsy is a chronic neurological disorder characterized by abnormal and synchronous overdischarge of neurons in the brain. It is one of the most common neurological disorders in the world, affecting approximately 50 million people (1, 2). With the intensification of global aging population, the incidence of geriatric epilepsy has shown a significant upward trend, and it has become the third most common neurological disease among the elderly, following stroke and dementia (3). Recent clinical evidence indicates that the global burden of idiopathic epilepsy in adults aged 60 and above increased by over 190% between 1990 and 2021, with prevalence rates projected to continue rising through 2035 (3). Despite its high prevalence, diagnosing epilepsy in older adults remains challenging as it frequently presents with atypical semiology rather than classic tonic-clonic movements (4). Older patients often exhibit focal seizures with altered awareness or non-convulsive status epilepticus (NCSE), which represents a prolonged state of seizures without convulsions. In the elderly, NCSE is the most common form of status epilepticus and can be easily misidentified as other age-related cognitive fluctuations or metabolic encephalopathy (4), often leading to unrecognized cases and delays in diagnosis and treatment. Clinical studies focusing on geriatric populations have demonstrated that the thirty-day mortality rate for NCSE is notably high, reaching approximately 26%. Specifically, the occurrence of comatose subtypes of NCSE serves as a robust predictor for poor outcomes, with the risk of death increasing significantly in patients who present with coma or those without a prior history of recurrent status epilepticus (5). Epileptic seizures usually may cause cognitive, neurological and physiological damage, and lead to loss of consciousness and sudden death. Through timely diagnosis and monitoring, these risks can be significantly reduced (6). Therefore, how to accurately and in real time detect epilepsy—especially these non-obvious seizure types—is of great significance for clinical treatment (7). Electroencephalogram (EEG) is widely used to monitor brain electrical activity and provide important information on the dynamics of epileptic seizures due to its non-invasive nature and high temporal resolution (8, 9).

To address these challenges, many automatic detection methods (10) have been explored. Traditional machine learning based methods usually rely on handcrafted functions (11). For instance, Akter et al. (12) employed statistical features in high-frequency bands, while Shen et al. (13) proposed a real-time detection approach using discrete wavelet transform. Both demonstrated excellent performance. However, recent deep learning models have significantly improved the performance of brain signal analysis. For instance, advanced architectures incorporating multi-head self-attention mechanisms (14), Transformer networks for high-frequency oscillation detection (15), and dual-domain feature extraction combined with generative adversarial networks (16) have achieved remarkable success. Despite these advances, most existing models are still limited by high data dependence, computational cost, and individual variability. The functional brain network provides a new approach to revealing synchronous and structural information by simulating the dynamic interactions between brain regions. Based on this, we propose a hybrid deep learning framework that integrates functional brain networks, termed TFFBN-HDLF. First, we design a time–frequency brain network construction method, TFFBNC, which combines PCC and PLV, comprehensively capturing synchronized brain activity across both time and frequency domains. Furthermore, we develop a hybrid deep learning architecture, SeizureTransNet, which integrates CNN with an enhanced Transformer module to dynamically select and fuse multi-scale spatiotemporal features.

The main contributions of this study are summarized as follows:

1. We propose a novel TFPPNet that integrates PCC and PLV in both the time domain and the frequency domain. This design can simultaneously capture dynamic synchronous and stable connection patterns, thereby enhancing the characterization ability of EEG features, which is used for the detection of epileptic seizures.
2. We have developed a hybrid deep learning framework that combines CNN with the SK Attention enhanced Transformer architecture. By taking advantage of the local feature extraction feature of CNN, the spatial dependency relationship between the channels of EEG signals is obtained. By combining the long-term modeling characteristics of Transformer and the adaptive selection of multi-scale features of SK attention, a more comprehensive spatiotemporal representation learning is achieved.
3. We propose an outstanding epilepsy detection method, which enhances its clinical applicability and generalization ability in different patient groups. Extensive experiments were conducted on two widely used public datasets, proving the effectiveness and robustness of the proposed method.

## 2 Related work

### 2.1 The study of functional brain network

In neurological diseases such as epilepsy, constructing complex functional brain networks and analyzing their network topological properties has become an important research paradigm. This method can quantify the strength of functional connections between brain regions and effectively discover the dynamic patterns of the brain under different physiological states. Early studies typically utilized phase synchronization methods to construct functional connection matrices in specific frequency bands in multi-channel EEG signals, and further employed graph theory indicators to characterize the integration and separation of brain networks. For instance, a study on neonatal epileptic seizures combined with functional brain networks found that during epileptic seizures, whole brain synchronization is significantly enhanced and exhibits a highly regularized topological structure (17). With the development of brain network theory, various topological features of brain networks have been extracted to enhance the discriminative ability of models in epilepsy detection. Supriya et al. (8) transforms EEG time series into complex networks, effectively capturing the structural differences of brain networks in different states of epilepsy and enhancing the accuracy of epilepsy detection. Furthermore, how to effectively extract the multi-scale periodic dynamic features exhibited by brain networks is of crucial importance. Recent studies (18, 19) have utilized this characteristic to enhance performance in epilepsy detection. These works indicate that epileptic seizures are controlled by internal biological rhythms on multiple levels. Meanwhile, significant progress has also been made in feature engineering based on the functional network structures of PLV (20, 21) and PCC (22).

In conclusion, the brain functional network has demonstrated strong potential in clarifying the mechanism of epilepsy and predicting epileptic seizures, and its form has evolved from traditional static topological analysis to the current multi-scale dynamic modeling. However, the fusion of multiple functional brain networks and the coordination mechanisms of different brain networks have not been fully articulated.

### 2.2 The study of seizure detection

In recent years, automatic seizure detection methods based on EEG signals have achieved significant progress (23). Early studies primarily relied on handcrafted time-frequency domain features combined with traditional classifiers. For instance, one study employed the discrete wavelet transform to perform multi-scale decomposition of EEG signals and extracted statistical features from each sub-band, achieving promising results (13). Additionally, another study combined filter banks with mutual information-driven feature optimization and obtained favorable performance on a dataset comprising data from 11 patients (12).

The development of deep learning has brought new solutions for automated epilepsy detection. For instance, a CNN-BiLSTM hybrid model was applied to epilepsy detection, effectively enhancing classification performance by combining the advantages of local feature extraction in convolutional layers and long-term dependency modeling in bidirectional long short-term memory networks (24). Furthermore, a stacked ensemble deep neural network based on ensemble learning patterns enhances robustness by integrating multiple fundamental models (25). By leveraging advanced strategies such as attention mechanisms and curriculum learning, the adaptability and generalizability of the model among patients can be further enhanced. For instance, the CABLNet model integrates convolutional layers, multi-head self-attention, and BiLSTM to effectively capture dynamic inter-channel dependencies and temporal context information (26). Similarly, SyncLearnNet proposes a generalized detection framework that extracts multi-scale variational features, captures the relationships between samples using the attention mechanism, and refines the classification boundaries in combination with curriculum learning, thereby enhancing model adaptability (27).

In summary, current research on epileptic seizure detection has achieved significant breakthroughs in feature extraction and model architecture. However, how to further improve the interpretability and generalization of the model across subjects remains a core challenge that urgently needs to be addressed.

## 3 Methods

### 3.1 Datasets

To evaluate the effectiveness of the method, two widely used publicly available scalp EEG datasets are used: Siena (28) and CHB-MIT (29). The CHB-MIT dataset, contains multi-channel EEG records of 23 patients aged 5 to 22. These data include approximately 940 hours of recording, sampled at 256 Hz, and channels 22–24 follow the international 10–20 system. A total of 198 episodes were included, mainly complex partial episodes, all with clinical annotations. The Siena dataset included EEG and ECG records of 14 adult patients with focal epilepsy (9 males and 5 females, with an average age of 43.5 years). It contains at least 19 standard channels with a total of approximately 144 h of EEG records, and the sampled at 512Hz. The average duration of 47 epileptic seizures was 61 seconds. These datasets cover different ages, epilepsy types and acquisition conditions, ensuring the diversity and complexity of the data. All experiments were conducted independently on each dataset to ensure objectivity and comparability.

### 3.2 The proposed TFFBN-HDLF framework

**Figure 1:**
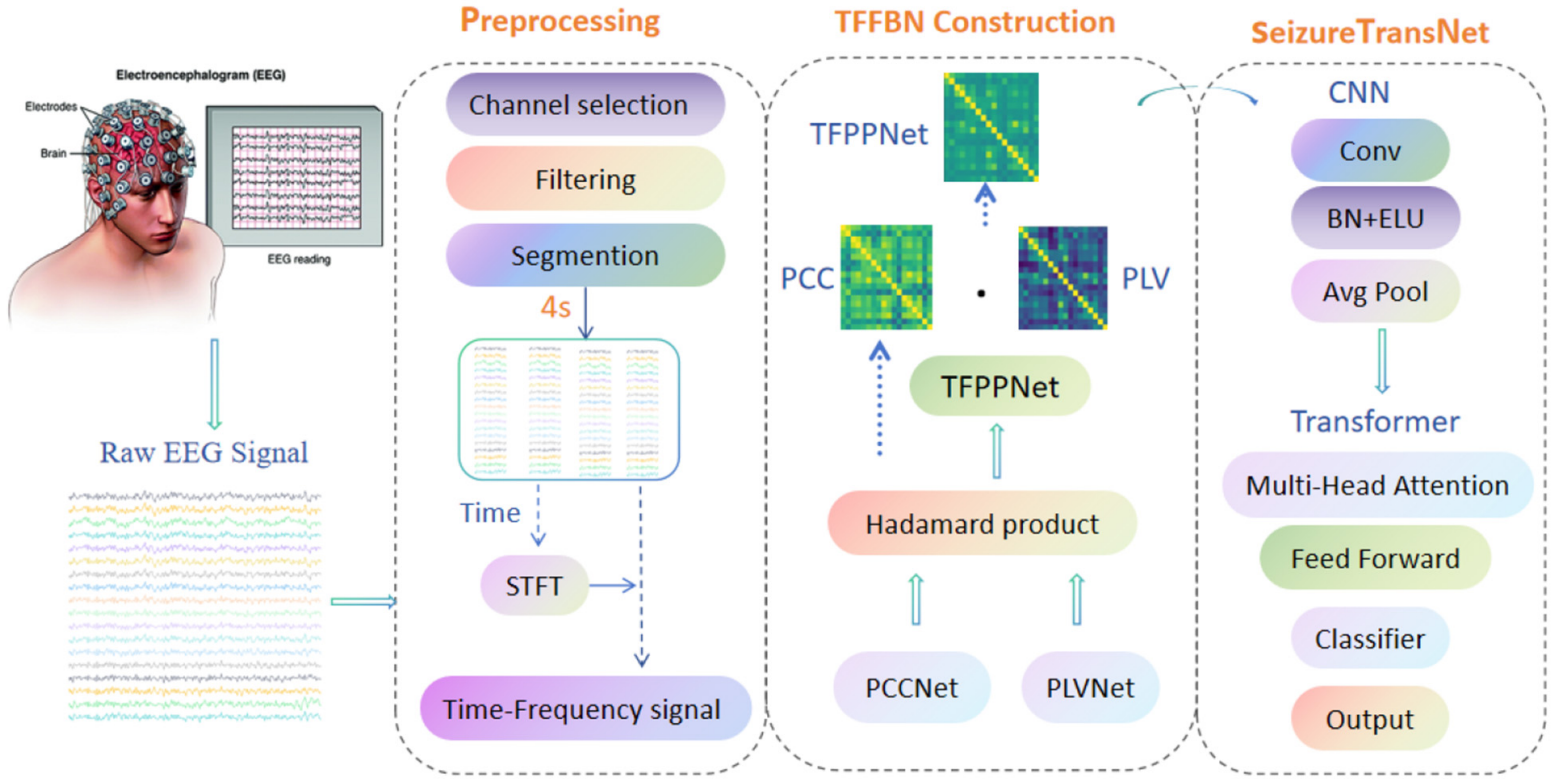
Overall framework of the proposed TFFBN-HDLF.

We propose a TFFBN-HDLF framework that integrates time-frequency functional brain networks with a hybrid deep learning model. The detailed pipeline is illustrated in Figure 1. First, the raw EEG data undergoes channel selection, filtering, and segmentation. To obtain frequency-domain information, the preprocessed data is transformed into a time-frequency representation using the Short-Time Fourier Transform (STFT). Subsequently, PCC and PLV based functional brain networks are computed and fused in the time-frequency domain to construct the integrated functional brain network, termed TFPP. Finally, the TFPP is fed into the SeizureTransNet hybrid deep learning model for epileptic seizure classification.

#### 3.2.1 Preprocessing

Complete preprocessing was carried out on the two datasets to ensure the reliability of model training and evaluation. Firstly, a bandpass filter of 1 to 50 Hz was applied to the original EEG signal. Then, a sliding window was used to split the signal into 4-second segments. This window length was selected to balance spectral stability with temporal sensitivity. A 4-second duration ensures at least four full cycles of the lowest frequency (1 Hz), allowing for reliable estimation of connectivity metrics like PCC and PLV, while remaining short enough to capture rapid seizure transitions. To address the scarcity of ictal samples, oversampling techniques are applied. To ensure channel consistency, 18 and 29 standard channels were selected for CHB-MIT and Siena datasets, respectively. Ultimately, these datasets were transformed into a consistent format suitable for brain network construction and deep learning analysis.

#### 3.2.2 Time-frequency functional brain network construction

##### 3.2.2.1 PCC construction

The PCC is utilized to evaluate the linear correlation between two EEG channels. The PCC between channel *X* and channel *Y* is calculated as follows (Equation 1):


$$\begin{array}{l} \rho_{X,Y}=\frac{\sum_{i=1}^{n}\left(x_{i}-\bar{X}\right)\left(y_{i}-Ȳ\right)}{\sqrt{\sum_{i=1}^{n}{\left(x_{i}-\bar{X}\right)}^{2}\sum_{i=1}^{n}{\left(y_{i}-Ȳ\right)}^{2}}} \end{array}$$ (1)

Here, *x*_*i*_ and *y*_*i*_ respectively represent the amplitudes of channels *X* and *Y* at the *i*-th sampling point, $\bar{X}$ and Ȳ respectively represent the corresponding mean values, and *n* represents the total number of sampling points. The stronger the linear synchronization of the two channels is, the greater the absolute value of the correlation coefficient ρ_*X, Y*_ will be. The PCC brain network represents the instantaneous coupling relationship between EEG signals and can effectively reflect the synchrony of brain regions.

##### 3.2.2.2 PLV construction

The PLV network represents the phase synchronization between channel pairs. By calculating the PLV values between each pair of EEG signal channels within the defined time window, a PLV brain network is ultimately formed. To calculate PLV, the Hilbert transform is first used to obtain the instantaneous phase of each EEG signal channel. Then evaluate the consistency of the phase difference angle distribution at each time point. The PLV element value ranging from 0 to 1 indicates the intensity of phase synchronization. The PLV between channels *x* and *y* is defined as Equation 2:


$$\begin{array}{l} {\text{PLV}}_{x,y}=\left|\frac{1}{N}\overset{N}{\underset{t=1}{\sum }}e^{j\left(\phi_{x}\left(t\right)-\phi_{y}\left(t\right)\right)}\right| \end{array}$$ (2)

Here, ϕ_*x*_(*t*) and ϕ_*y*_(*t*) represent the instantaneous phases of channels *x* and *y* at time *t*, *N* is the total number of sampling points, and *j* is the imaginary unit. The higher the PLV value, the more stable the phase difference it reflects, representing the phase synchrony of brain regions. By evaluating the phase consistency of the entire observation window, PLV effectively captures the coordinated neuronal activity associated with epileptic seizures.

##### 3.2.2.3 Brain network fusion

A hybrid brain functional network representation (TFPP) was designed to effectively integrate the amplitude coupling and phase synchronization characteristics of EEG signals. To emphasize inter-channel positive correlations while mitigating the confounding effects of anti-correlations, we compute the absolute value of the PCC matrix. Subsequently, the Hadamard product is applied to fuse the PCC matrix with the PLV matrix within the same time window, yielding a Phase-Correlation (PP) matrix that jointly encodes amplitude and phase information.

To capture the interaction between amplitude- and phase-based connectivity, we utilize this Hadamard product to generate the PP matrix. Conceptually, this entry-wise multiplication serves as a synergistic integration mechanism: it explicitly models cross-domain coupling by assigning high weight only to functional connections where signal intensity fluctuations are temporally coordinated with phase consistency. Unlike traditional methods like feature concatenation—which treat connectivity measures as independent vectors—this fusion strategy enforces a joint dependency at the network edge level. By filtering the brain network through this dual-constraint approach, the subsequent SeizureTransNet can more effectively extract subtle spatiotemporal features essential for detecting complex seizures, particularly in geriatric or non-convulsive status epilepticus (NCSE) cases.

To further capture dynamic characteristics across both time and frequency domains, we generate frequency-domain representations using the Short-Time Fourier Transform (STFT) and compute the corresponding PP matrices for each frequency band. All PP matrices derived from multiple time windows and frequency bands are then concatenated in a predefined order to construct the final TFPP tensor.

Therefore, the TFPP representation provides a unified integration of multi-dimensional EEG features, capturing the dynamic evolution of brain functional networks across time and frequency. This rich spatiotemporal characterization enhances the discriminative capacity of the neural representation, thereby improving both accuracy and robustness in epileptic seizure detection—especially for atypical and challenging clinical presentations.

#### 3.2.3 The proposed SeizureTransNet model

**Figure 2:**
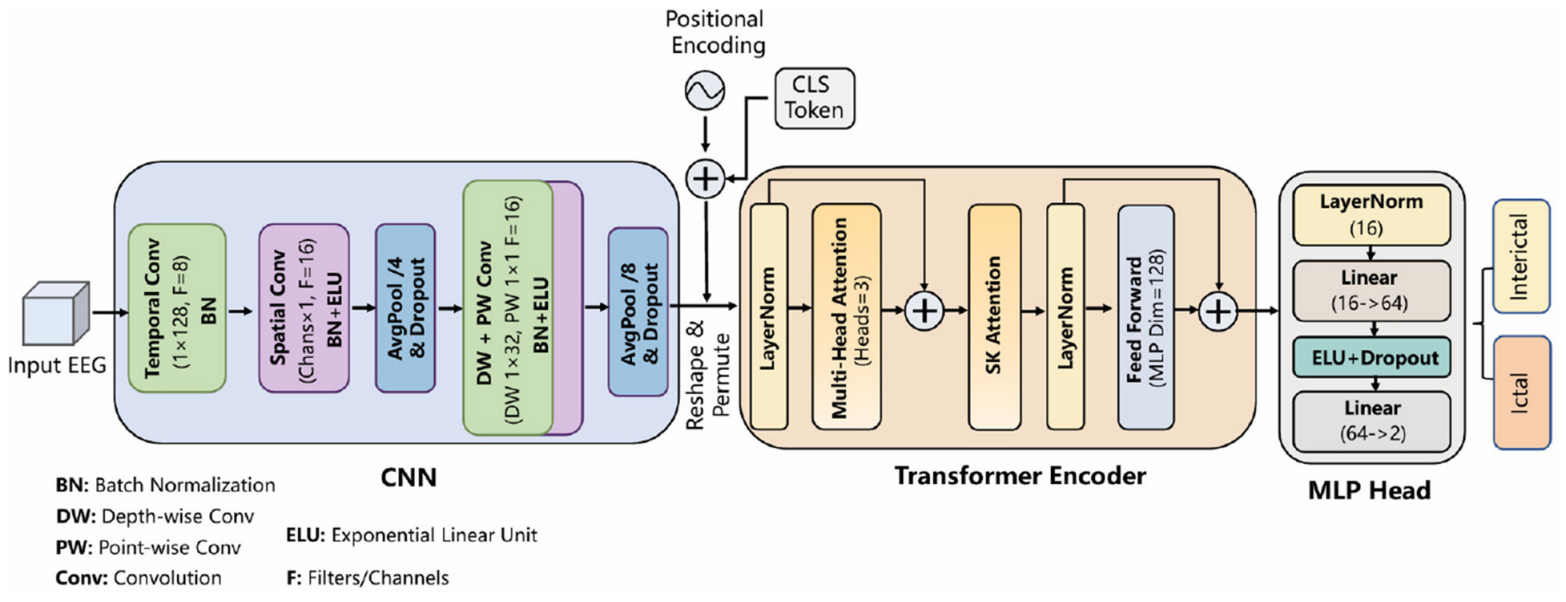
Overall framework of SeizureTransNet.

A hybrid architecture, SeizureTransNet, is proposed for epileptic seizure detection. This architecture employs a CNN module to extract local spatiotemporal features from EEG signals and utilizes a Transformer enhanced with SK attention to capture global contextual dependencies. Finally, an MLP classifier is used to distinguish between different EEG states. The overall architecture of SeizureTransNet is illustrated in Figure 2.

A CNN module serves as the Temporal-Spatial Feature Extractor. The CNN consists of a temporal convolution layer, a spatial convolution layer, and two Depthwise Separable Convolution layers. First, the raw EEG data passes through a temporal convolution layer to extract dynamic temporal patterns. Next, a spatial convolution layer captures the dependencies among electrode channels. Subsequently, two layers of depthwise separable convolutions are applied to reduce model complexity and effectively improve computational efficiency. Between each layer, average pooling and dropout are employed for feature dimensionality reduction and regularization, respectively.

An enhanced Transformer module is used to capture global context information and strengthen the feature representation of EEG signals. The features extracted by the CNN module are first reshaped and positionally encoded. In addition, a learnable class token is added to the sequence to retain global information. The enhanced Transformer encoder block combines multi-head attention and SK (Selective kernel) attention mechanisms. The global dependency of the entire sequence is modeled through multi-head attention, while SK attention is used to adaptively weight the multi-scale spatiotemporal information. This combined architecture enables the model to have both the ability to extract multi-scale features and capture long-range global dependencies.

Finally, the feature representations through the Transformer are input into an MLP for the final classification of Ictal and Interictal. MLP consists of a standard normalization layer, a linear projection layer, an ELU activation function, dropout, etc.

## 4 Experiments

### 4.1 Experimental setup and evaluation metrics

The model was evaluated using five commonly used metrics: accuracy (Acc), sensitivity (Sen), specificity (Spe), and area under the curve (AUC) (Equations 3–5).


$$\begin{array}{l} \text{Acc}=\frac{TP+TN}{TP+TN+FP+FN} \end{array}$$ (3)

$$\begin{array}{l} \text{Sen}=\frac{TP}{TP+FN} \end{array}$$ (4)

$$\begin{array}{l} \text{Spe}=\frac{TN}{TN+FP} \end{array}$$ (5)

Here, TP, TN, FP and FN respectively represent true positive, true negative, false positive and false negative. Acc represents the overall correctness of the model. Sen evaluated the ability of the model to correctly identify epileptic seizures.Spe reflects the model's ability to resist false alarms. AUC is derived from the ROC curves of TPR (true positive rate) and FPR (false positive rate), providing a balanced measure that can effectively and fairly represent the performance of the model even on imbalanced datasets.

Ten-fold cross validation is adopted to ensure the robustness of the model. The result metric of the final model is the average of all folds. All experiments were implemented based on the platform of Python 3.8, PyTorch 1.12 and NVIDIA A100 GPU. The Adam optimizer was used for training, with an initial learning rate of 0.001 and a batch size of 256. To prevent overfitting, an early stop policy was adopted, and the patience value was set to 10 epochs.

## 5 Results and discussion

### 5.1 Performance on CHB-MIT

**Figure 3:**
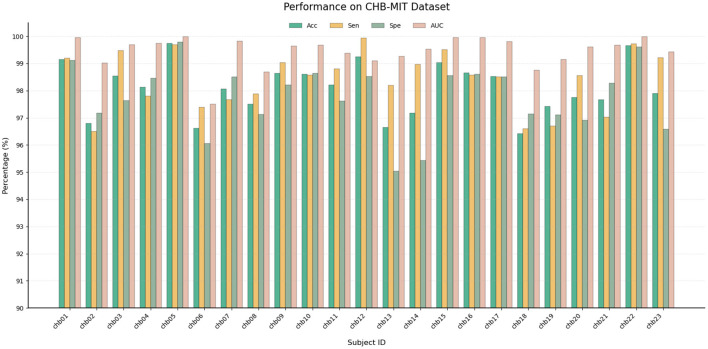
Performance on the CHB-MIT dataset.

The model results on the CHB-MIT Dataset are shown in Figure 3 and Table 1. The proposed model achieved outstanding performance, obtaining 98.09% Acc, 98.41% Sen, 97.76% Spe, and 99.45% AUC. The subjects chb05 and chb22 might have achieved good results due to their high-quality EEG records, with Acc values approaching 100%. In addition, the Spe values of subjects chb01 and chb05 reached 99.11% and 99.79% respectively, reducing a large number of false positives and proving the robustness of the model. On the other hand, the Sen scores of chb06 and chb18 were at relatively low levels, possibly because some of the epileptic seizures in these subjects exhibited strong physiological specificity, making them difficult to capture with a unified model. Despite this, the AUC scores of all subjects were above 0.97, confirming that the model has a strong discriminatory ability for the detection of epileptic seizures. Although the scores of a few subjects slightly declined, the overall scores remained at a high level, reflecting stable performance and strong generalization ability.

**Table 1:** Experimental results based on the CHB-MIT dataset.

**ID**	**Acc(%)**	**Sen(%)**	**Spe(%)**	**AUC(%)**
chb01	99.15	99.19	99.11	99.96
chb02	96.79	96.50	97.17	99.01
chb03	98.54	99.47	97.63	99.69
chb04	98.12	97.80	98.46	99.74
chb05	99.74	99.69	99.79	99.98
chb06	96.62	97.39	96.06	97.51
chb07	98.07	97.66	98.50	99.82
chb08	97.50	97.88	97.12	98.69
chb09	98.63	99.03	98.21	99.64
chb10	98.60	98.57	98.64	99.67
chb11	98.21	98.81	97.62	99.38
chb12	99.25	99.93	98.53	99.10
chb13	96.64	98.19	95.04	99.26
chb14	97.17	98.97	95.43	99.52
chb15	99.04	99.51	98.56	99.96
chb16	98.66	98.58	98.61	99.96
chb17	98.52	98.51	98.51	99.81
chb18	96.41	96.60	97.14	98.76
chb19	97.42	96.69	97.10	99.14
chb20	97.75	98.55	96.91	99.60
chb21	97.66	97.02	98.27	99.67
chb22	99.66	99.73	99.60	99.99
chb23	97.90	99.22	96.58	99.42
**AVG**	**98.09**	**98.41**	**97.76**	**99.45**

Bold values indicate the best performance metrics.

### 5.2 Performance on Siena

**Figure 4:**
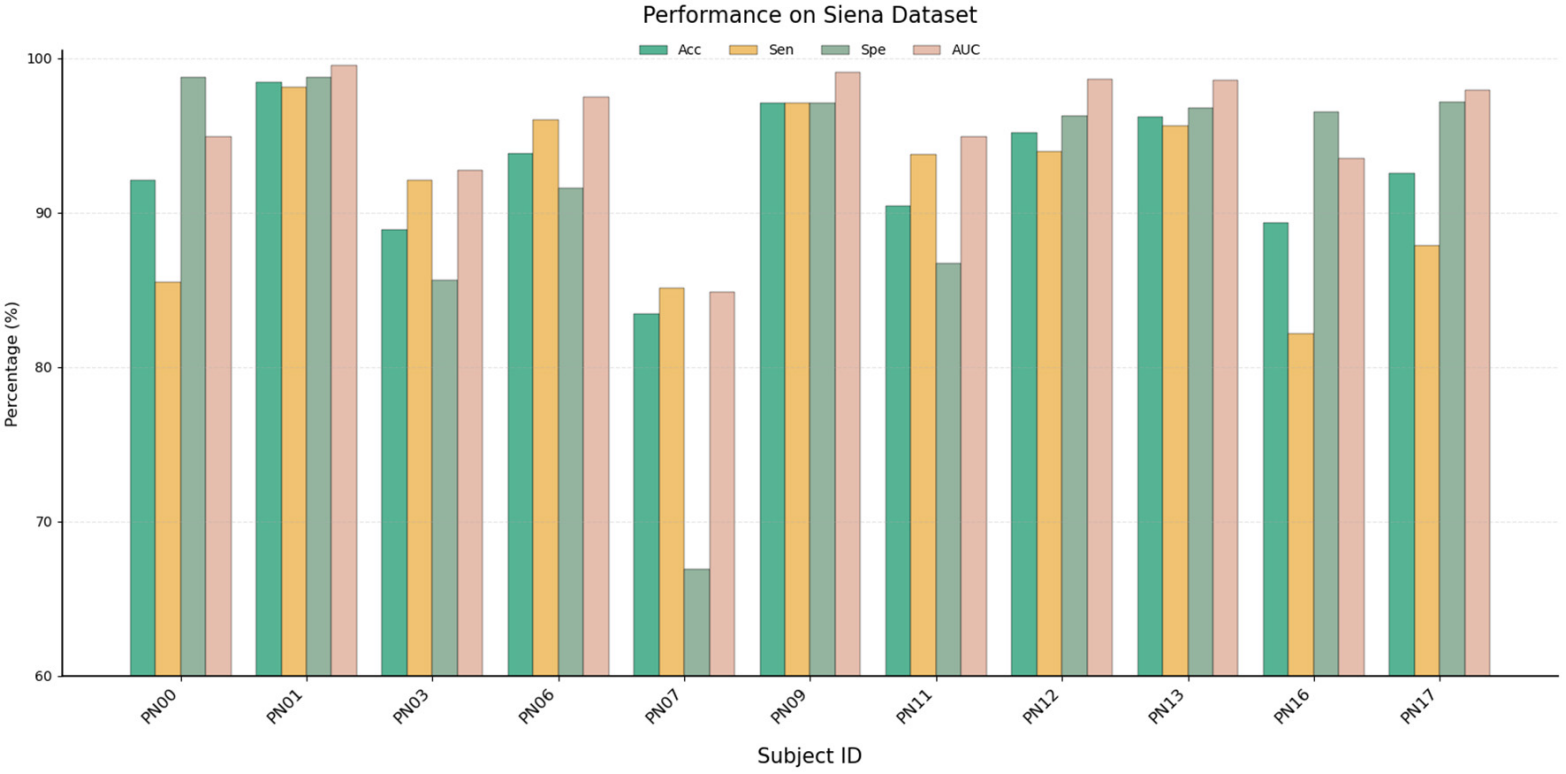
Performance on the Siena Dataset.

The results of the model on the Siena Dataset are shown in Figure 4 and Table 2. The proposed model still achieved strong performance, reaching 92.49% Acc, Sen 92.93%, Spe 92.02%, and 95.64% AUC. Subjects PN01 and PN09 achieved relatively high Acc, which were 98.42% and 97.06% respectively, demonstrating the powerful detection capability of the model. The sensitivity of PN07 and PN16 was shown to be relatively low, possibly due to the scarcity of seizure samples in these subjects. Meanwhile, the Spe values of PN00, PN01 and PN17 are all above 97%, significantly reducing the number of false alarms. Although the overall performance of the Siena dataset may be slightly lower than that of CHB-MIT due to its smaller sample size. However, similar to CHB-MIT, most of the participants still achieved relatively good results. These results prove that the model has a strong generalization ability across different datasets.

**Table 2:** Experimental results based on the Siena dataset.

**ID**	**Acc(%)**	**Sen(%)**	**Spe(%)**	**AUC(%)**
PN00	92.10	85.49	98.74	94.93
PN01	98.42	98.09	98.75	99.53
PN03	88.89	92.11	85.64	92.71
PN06	93.79	96.00	91.57	97.49
PN07	83.47	85.10	66.94	84.82
PN09	97.06	97.12	97.11	99.09
PN11	90.43	93.74	86.72	94.90
PN12	95.14	93.97	96.28	98.64
PN13	96.22	95.64	96.78	98.54
PN16	89.35	82.17	96.52	93.49
PN17	92.52	87.87	97.16	97.90
**AVG**	**92.49**	**92.93**	**92.02**	**95.64**

Bold values indicate the best performance metrics.

### 5.3 Ablation study

As shown in Table 3, when only the original EEG was used, the Acc of this model was 97.76%, the Sen was 97.89%, and the Spe was 97.60%. After combining the features of PCC and PLV, the performance gradually improved. The method incorporating PCC features achieved 97.84% Acc, 98.14% Sen, and 97.61% Spe. Meanwhile, the method incorporating PLV features achieved 97.89% Acc, 98.16% Sen, and 97.66% Spe. The TFPP fusion achieved the best effect: Acc was 98.09%, Sen was 98.41%, and Spe was 97.76%. Compared to using single-feature information alone, the TFPP fusion, which integrates multiple features, better captures the differences between distinct brain states.

**Table 3:** Ablation results for different functional brain networks.

**Input signal**	**Acc (%)**	**Sen (%)**	**Spe (%)**	**AUC (%)**
EEG	97.76	97.89	97.60	98.82
EEG+PCC	97.84	98.14	97.61	99.01
EEG+PLV	97.89	98.16	97.66	99.18
**EEG+TFPP**	**98.0**6	**98.4**1	**97.76**	**99.45**

Bold values indicate the best performance metrics.

The results show that functional brain networks enrich feature representation capabilities by integrating spatial connection information between brain regions. While retaining the time-frequency features, this method enhances the discriminative performance of the model and verifies the effectiveness of the proposed multimodal fusion strategy for epileptic seizure detection.

### 5.4 Visualization of functional brain networks

**Figure 5:**
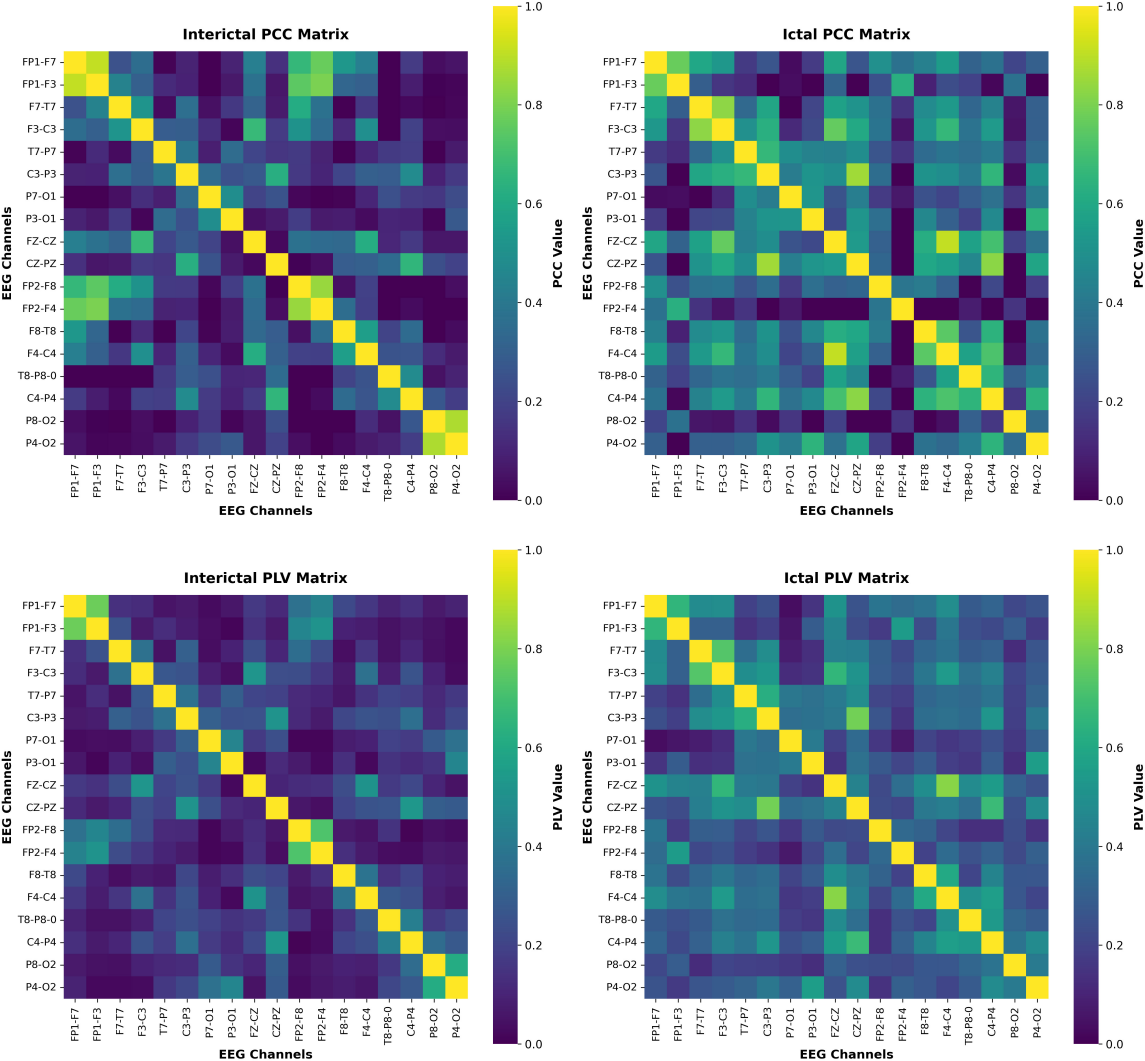
The visualization results on the CHB-MIT.

**Figure 6:**
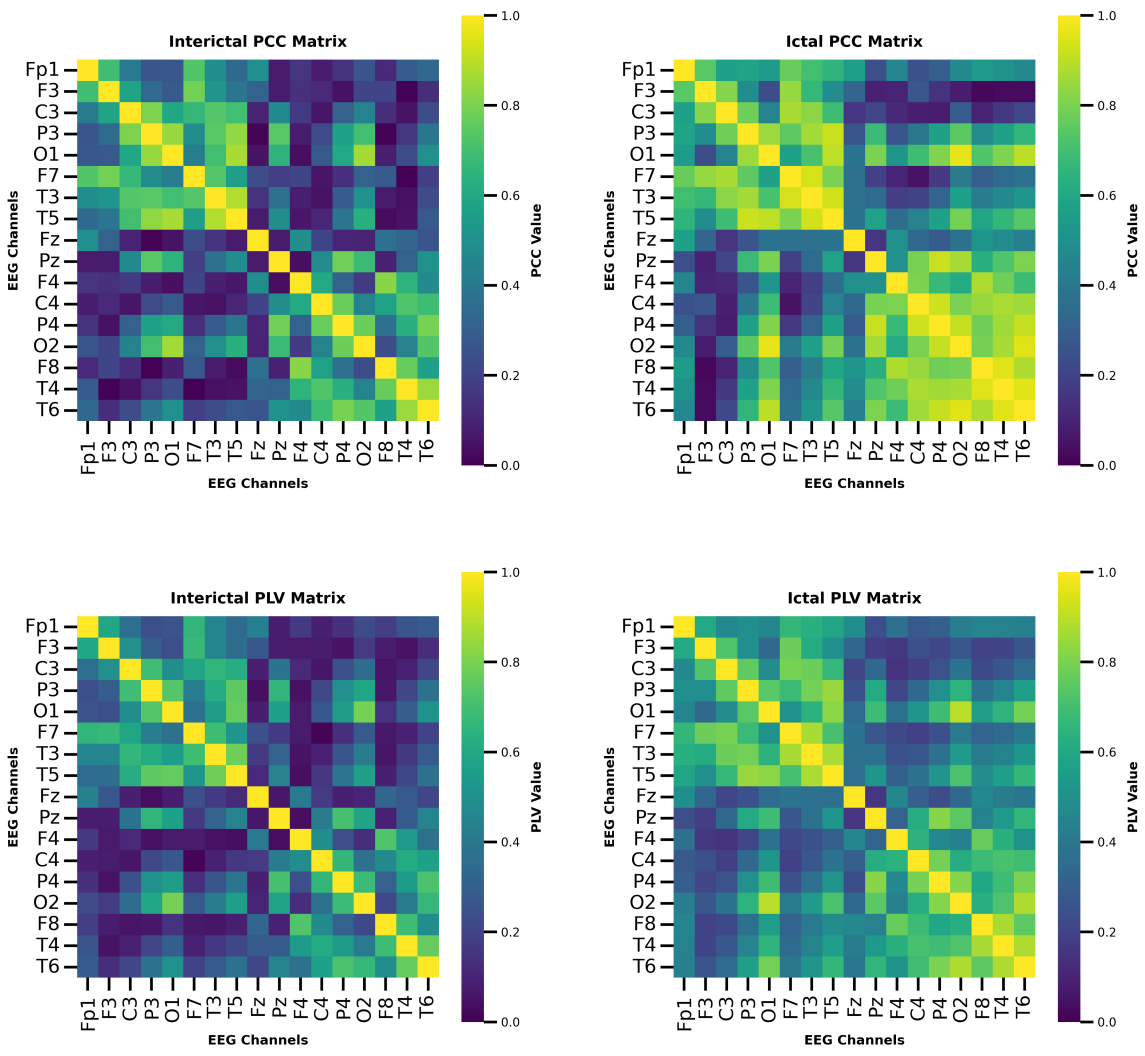
The visualization results on the Siena.

To analyze the dynamic changes of neural connections during the ictal period and the interictal period, the PCC and PLA brain networks were visualized. In order to effectively analyze the overall trend of brain network changes, samples with moderate performance in classification tasks were selected. By analyzing the brain functional connection patterns, a deeper understanding of the relationship between input features and model decisions can be achieved. The visualization results of the CHB-MIT and Siena datasets are shown in Figures 5, 6, respectively.

By combining PCC and PLV brain networks, a complementary understanding of brain networks is provided. The PCC reflects the synchronization relationship of amplitude between channels, while the PLV matrix reflects the synchronization relationship of phase. These features show a consistent trend in the two datasets. In the interictal states, the PCC and PLV brain networks exhibit relatively weak and sparse connections, indicating that the connections between brain regions are weak under normal brain activity. However, in the ictal state, the global connectivity of the PCC and PLV brain networks significantly increases. Furthermore, a stronger correlation emerged between channels at greater distances, indicating extensive synchrony. This synchronous pattern is consistent with previous findings that epileptic seizures enhance connectivity between different regions of the brain. This is mainly because epileptic seizures usually involve abnormal electrical activity distributed across multiple brain regions.

Overall, by visualizing the PCC and PLV brain networks, the dynamic changes between brain networks in different states were demonstrated, enhancing the interpretability of the model during decision making. In addition, by analyzing the dynamic changes in brain networks, the understanding of the internal mechanism of epileptic seizures has been deepened.

### 5.5 Performance comparison with baseline models

Under a unified experimental setup, the performance differences between the proposed SeizureTransNet and multiple baseline models were comprehensively evaluated on two benchmark datasets. These baselines include CABLNet, GCN-BiGRU, TCN-BiLSTM and WT-RNN. The detailed comparison results are shown in the Table 4.

**Table 4:** Performance comparison with baseline models on the two datasets.

**Dataset**	**Model**	**Acc (%)**	**Sen (%)**	**Spe (%)**	**AUC (%)**
CHB-MIT	CABLNet (26)	97.12	96.56	97.08	99.15
	GCN-BiGRU (22)	95.71	96.59	95.65	98.95
	TCN-BiLSTM (30)	95.54	94.77	96.29	98.76
	WT-RNN (31)	94.34	93.56	96.60	98.55
	**SeizureTransNet**	**98.09**	**98.41**	**97.76**	**99.45**
Siena	CABLNet (26)	91.75	91.54	91.83	95.25
	GCN-BiGRU (22)	90.18	91.61	90.98	94.40
	TCN-BiLSTM (30)	91.26	90.54	91.95	94.90
	WT-RNN (31)	90.63	90.41	91.56	94.15
	**SeizureTransNet**	**92.49**	**92.93**	**92.02**	**95.64**

Bold values indicate the best performance metrics.

On the CHB-MIT dataset, SeizureTransNet achieved the best performance metrics compared to other baseline models, reaching 98.09% Acc, 98.41% Sen, 97.76% Spe, and 99.45% AUC. Among them, WT-RNN achieved the worst results, possibly because it has the advantage of modeling long sequences, but fails to capture the time domain features from short sequences well. CABLNet achieves effective temporal feature extraction through the multi-head attention mechanism, and its performance is superior to traditional models such as TCN-BiLSTM and GCN-BiGRU. However, it is unable to extract features in the frequency domain. In contrast, SeizureTransNet integrates time domain and frequency domain features, thereby overcoming the limitations of relying on a single type of feature representation, which enables this method to achieve optimal performance.

Furthermore, SeizureTransNet still achieved the best results on the Siena dataset, although on a smaller scale compared to the CHB-MIT dataset. It achieved 92.49% Acc, 92.93% Sen, 92.02% Spe, and 95.64% AUC. GCN-BiGRU selectively captures data features through graph learning and gating mechanisms to enhance the ability of epilepsy detection, and its sensitivity is higher than that of TCN-BiLSTM and WT-RNN. Moreover, due to the advantages of the self focus mechanism, CABLNet consistently maintains high performance in the Siena dataset.

The consistency of the experimental results observed on the two benchmark datasets indicates that the proposed SeizureTransNet model not only performs well on large-scale data, but also demonstrates strong stability and generalization ability in small-sample clinical scenarios. Overall, the results demonstrated that SeizureTransNet achieved excellent and balanced classification performance across different datasets and baseline comparisons, verifying its robustness and clinical applicability.

## 6 Conclusion

A TFFBN-HDLF framework for epileptic seizure detection has been proposed. By combining the novel brain network construction method TFPP and the efficient hybrid deep learning model SeizureTransNet, the time-frequency characteristics of EEG signals can be effectively captured, enabling accurate identification of epileptic seizure states and providing an efficient solution for clinical decision-making.

By combining the inter-channel synchronization information of the PCC brain network and the inter-channel phase dynamics of the PLV brain network, a unified functional brain network representation TFPP is generated. Furthermore, in order to learn effectively from this representation, a hybrid architecture combining CNN and SK attention enhanced Transformer is proposed, SeizureTransNet. The CNN component effectively extracts spatiotemporal features, while the Transformer utilizes the self-attention mechanism to effectively model long-term temporal dependencies. By combining the advantages of the two structures, the model's ability to learn spatiotemporal features has been significantly enhanced, and its generalization ability has been improved. Extensive experiments conducted on the public CHB-MIT and Siena datasets have demonstrated the superior performance of the proposed framework and achieved state-of-the-art results. These results indicate that combining functional brain networks with advanced deep learning architectures can effectively address the issue of poor performance in epilepsy detection.

Despite these achievements, a detailed analysis of cases with poorer performance reveals certain limitations of the current framework. Misclassifications were observed to occur primarily in EEG segments heavily contaminated by intense myogenic artifacts (e.g., subject PN07 in the Siena dataset), which can distort functional connectivity estimation. Additionally, relatively lower sensitivity in specific subjects (e.g., chb06 and chb18) suggests that seizure patterns with strong physiological specificity or ultra-low voltage characteristics remain challenging for a unified model. These findings are particularly significant given that geriatric epilepsy often presents with atypical or non-convulsive semiology, which leads to high thirty-day mortality rates and diagnostic delays. Future work will focus on integrating advanced automated artifact removal and adaptive fusion strategies to further enhance the model's reliability in complex clinical environments.

Overall, we have proposed a new framework for detecting epileptic seizures. This framework not only enhances the detection performance but also has potential clinical applicability, which is conducive to more effective real-time monitoring and support for computer-assisted medical diagnosis.

## Data Availability

The datasets presented in this study can be found in online repositories. The names of the repository/repositories and accession number(s) can be found below: The CHB-MIT v1.0.0 datasets are used during this work and are publicly available at: https://physionet.org/content/chbmit/1.0.0/. The Siena v1.0.0 datasets are used during this work and are publicly available at: https://physionet.org/content/siena-scalp-eeg/1.0.0/.
